# Veterinary Medical Association of New Jersey

**Published:** 1884-07

**Authors:** J. Gerth

**Affiliations:** Secretary


### VETERINARY MEDICAL ASSOCIATION OF NEW JERSEY.

*Organization Meeting.*

The above named Association organized at the office of Dr. James C. Corlies, No. 240 Market street, Newark, N. J., on Tuesday, Feb. 5, 1884. Fifty postal cards were mailed to veterinarians of this State, notifying them of the intention of organizing a State Veterinary Medical Association. Twenty veterinarians responded to the call.

At 2:15 P. M., J. Gerth, Jr., D.V.S., called the meeting to order. Dr. Corlies was elected temporary chairman, and the Association organized with the election of the following officers :

President—J. C. Corlies, D.V.S., of Newark.

Vice-Presidents—C. Lawrenz, V.S., Newark; Wm. B. E. Miller, D.V.S., Camden ; C. K. Dyer, V.S., Mount Holly.

Secretary—J. Gerth, Jr., D.V.S., Newark.

Cor. Secretary—J. H. Dancer, V.S., Orange.

Treasurer—A. Sherk, V.S., Newark.

Board of Censors—T. B. Rogers, D.V.S., Camden ; L. R. Sattler, V.S., Newark; D. J. Dixon, D.V.S., Hoboken; W. H. Arrowsmith, D.V.S., Jersey City; Wm. G. Schmidt, D.V.S., Newark.

Dr. W. H. Arrowsmith moved that this Association be known as the "Veterinary Medical Association of New Jersey." This motion was seconded and adopted.

A motion was made by Dr. J. Gerth, Jr., that the President appoint all committees, which motion was amended that not more than three members be appointed on each committee. Carried with amendment.

Dr. Dixon moved that the President call the next meeting at an early date. Seconded and adopted.

Meeting adjourned. J. Gerth, Jr., D.V.S., Sec.

*First Regular Meeting.*

The first regular meeting of the above named Association was held at Kleb's Hotel, Broad street, Newark, N. J., April 30, 1884. The President, Dr. J. C. Corlies, called the meeting to order at 3 P. M. Fourteen members were present. The minutes of the organization meeting were read and adopted.

Dr. Dixon remarked that before proceeding to any other business, it was necessary to first decide upon the adoption of the Constitution and By-Laws.

Dr. Miller moved that the report of the Committee on Constitution and By-Laws be received, ordered read, and adopted by part. Seconded and carried.

Dr. Corlies, the President, instructed the chairman of said Committee to proceed to the reading of the report.

Several sections of the Constitution and By-Laws were amended, and adopted as corrected.

The Association having adopted the Constitution and By-Laws, Dr. Dixon stated that it was now in order to proceed to the election of permanent officers for the ensuing year. A motion to that effect was made, seconded, and carried.

Drs. Miller, Dixon, and Corlies were nominated for President. Dr. Wm. B. E. Miller of Camden was declared elected.

Drs. C. K. Dyer of Mount Holly, and D. J. Dixon of Hoboken, were elected Vice-Presidents by acclamation.

Drs. H. W. Rowland of Jersey City, and J. Gerth, Jr., of Newark, were nominated for Secretary, and J. Gerth, Jr., elected.

Dr. W. P. Humphreys of Elizabeth was elected Treasurer.

Drs. J. C. Corlies, T. B. Rogers, H. W. Rowland, L. R. Sattler, and A. S. Leatherman were nominated to constitute the Board of Censors. All were elected by acclamation.

Dr. Corlies, before retiring, appointed a committee of two to escort the newly elected President to the chair, and thanked the Association for having conferred upon him the honor to preside over the organization of this Society, the first of its kind in our State.

Dr. Miller, on taking the chair, addressed the Association with a few appropriate remarks, and thanked the members for the honor of being elected their first permanent President.

After these few remarks the Association proceeded to business, and Dr. Corlies arose to a question of privilege, which was granted. He severely censured the State Board of Health of New Jersey for improperly conducting the stamping out of contagious pleuro-pneumonia, accused them of employing incompetent persons to act as their agents, and of illegally collecting fees for services rendered by their agents, and condemned their method of inoculation, etc. Dr. Corlies then introduced a resolution to that effect, requesting the Association to act upon it, and to endorse or reject it as they saw fit.

After considerable discussion, it was moved that a committee of three be appointed by the chair to carefully consider Dr. Corlies's communication and resolution, and report at the next meeting. Seconded and adopted.

The chair appointed Drs. Dixon, Rogers, and Rowland as a committee to consider the above communication, and report.

President Dr. Miller appointed Dr. T. B. Rogers and H. W. Rowland essayists for the next regular meeting, which will be held at Atlantic City, Aug. 14, 1884.

J. Gerth, Jr., D.V.S., Secretary.

